# Incidence and predictors of onboard injuries among Sri Lankan flight attendants

**DOI:** 10.1186/1471-2458-9-227

**Published:** 2009-07-11

**Authors:** Suneth B Agampodi, Samath D Dharmaratne, Thilini C Agampodi

**Affiliations:** 1 Department of Community Medicine, Faculty of Medicine and Allied Sciences, Rajarata University of Sri Lanka, Saliyapura, Sri Lanka; 2 Department of Community Medicine, Faculty of Medicine, University of Peradeniya, Sri Lanka

## Abstract

**Background:**

Occupational injuries among flight attendants have not been given appropriate attention in Sri Lanka. The purpose of this study was to estimate the incidence of onboard injury among Sri Lankan flight attendants and to describe the determinants of onboard injury.

**Methods:**

A descriptive cross-sectional study was carried out among Sri Lankan flight attendants. All flight attendants undergoing their annual health and first aid training were invited to participate. Flight attendants who flew continuously for a six-month period prior to data collection were included in the study sample. Recall history of injuries for a period of six months was recorded.

**Results:**

The study sample consisted of 98 (30.4%) male and 224 (69.6%) female flight attendants. The mean age of the study sample was 31 years (SD = 8) and the average duration of service was 10 years (SD = 7). A total of 100 onboard falls, slips or trips in the previous six months were reported by 52 (16.1%) respondents. Of the total sample, 128 (39.8%) cabin crew members reported an injury in the six months preceding the study. This represents a total injury incidence of 795 per 1000 person per year. The leading causes of injury was pulling, pushing or lifting (60.2%). The commonest type of injuries were strains and sprains (52.3%). Turbulence related injuries were reported by 38 (29.7%) flight attendants. The upper limbs (44.5%) and the back (32%) were the commonest sites affected. After controlling for other factors, female flight attendants had 2.9 times higher risk (95% CI 1.2–7.2) of sustaining and injury than males. Irrespective of sex, body weight less than 56 kilograms (OR 2.9, 95% CI 1.4–5.8) and less than seven years of on board experience (OR 10.5, 95% CI 3.6–31.0) were associated with higher risk of injury.

**Conclusion:**

Work related injury is a major occupational hazard to flight attendants. Appropriate preventive strategies are required to minimize them.

## Background

The presence of flight attendants are a mandatory requirement for all airlines to provide safety and security for passengers[1]. With the expansion of the airline industry and global travel, demand and opportunities for flight attendants have expanded globally in recent years. In Sri Lanka, around 3,000 flight attendants are currently employed by national and international airlines.

Working in a moving aircraft subjects flight attendants to high-risk of injury. Handling heavy luggage, overhead bins, service trolleys and air stair doors also increase the risk of injury during working. In addition, conditions such as turbulence make the onboard environment more dangerous.

All flight attendants must be certified by relevant aviation authorities of a country. This certification requires training in health, safety and various procedures which aim to minimise onboard injuries. Despite extensive training, flight attendants often suffer work related injuries. Many studies around the world have reported different injury rates ranging from 8.6 to 13.0 per 1000 full-time workers[2]. It is evident that injury rates based on registers, workers' compensation reports and health facility reports systematically underestimate the true injury incidence. Specifically, minor injuries are often not reported and not registered in a routine manner.

A major limitation of the existing findings is that the majority of them are from developed countries [3-8], whereas research concerning flight attendants from developing countries is scarce. Injury pattern, reporting and sequel might be different between developed and developing countries. They might also differ depending on the airline for which they work, compensation procedures, legal requirements and available medical facilities. Data on these factors is essential to launch an injury prevention program in this vulnerable occupational category.

In Sri Lanka, there are no published studies on injuries to flight attendant and health. Occupational injury contribute to a major portion of the global injury burden [9], and it is important to know the local burden of injury in this group. The purpose of the present study was to estimate the injury incidence among Sri Lankan flight attendants and to describe the determinants of onboard injuries.

## Methods

A descriptive cross sectional study was carried out among Sri Lankan flight attendants who attended their annual health and first aid training during a two-year period between 2007 and 2008. All flight attendants attending these training sessions were invited to participate in the study. Flight attendants undergoing initial training before their first flight (new recruits) and flight attendants who had not flown for six months (e.g., after childbirth) were excluded from the study.

The total study population consisted of 3,000 flight attendants. Required sample size was calculated to estimate an injury incidence within 10% of its true value and a 90% confidence level. A total of 271 respondents were required and with an expected 10% non-respondent rate, the required size of the sample was 298.

All consecutive flight attendants registering for training during the study period and fulfilling eligibility criteria were selected as study units. Informed verbal consent was obtained from all participants prior to data collection. Ethical clearance for the study was obtained from the Ethical Review Committee of the Faculty of Medicine, University of Peradeniya.

A self-administered questionnaire was used for data collection. Authors explained the study objectives and the questionnaire to the eligible subjects and collected data before each training session.

Data was managed and analyzed using SPSS software version 13. Descriptive statistics, percentages and proportions were used to present baseline data. Incidence rates were calculated for 1,000 person years. Predictors of onboard injuries were assessed using a logistic regression model. In this model, we first analyzed the unadjusted odds ratios for all probable predictors. Only the predictors that had a *p *value less than 0.2 were included in the final model.

## Results

### Characteristics of the study sample

Of the 326 selected flight attendants, 324 completed the questionnaire (response rate – 99.4%). When the flight attendants who did not fly during the previous six months were excluded, 322 questionnaires were eligible for analysis. The age of the respondents ranged from 21 years to 54 years. Table 1 shows the characteristics of the study sample.

**Table 1:** Characteristics of the study sample of 322 Sri Lankan cabin crew members

**Characteristic**		
Mean Age (SD)	31	(8)

Sex		
Male N (%)	98	(30.4%)
Female N (%)	224	(69.6%)

Ethnicity		
Sinhalese N (%)	222	(68.9%)
Tamil N (%)	34	(10.6%)
Moor/Malay N (%)	38	(11.8%)
Berger N (%)	28	(8.7%)

Mean duration of service (SD)	10	(7)

Mean Height in cm (SD)	166.4	(9.4)

Mean weight in kg (SD)	62.2	(13.7)

Ergonomic training		
Received N (%)	90	(28%)
Not received N (%)	232	(72%)

*Mean and SD values for age and duration of service is given in years

### Onboard falls, slips and trips

A total of 100 onboard falls, slips or trips in the previous six months were reported by 52 (16.1%) flight attendants. Of them, 22 (42.3%) experienced it once, 24 (46.2%) twice and six (11.5%) more than two times.

### Onboard injuries

Of the total sample, 128 (39.8%) cabin crewmembers reported an onboard injury in the six months preceding the study, representing a total injury incidence of 795 per 1000 person years.

Details of the most severe onboard injury reported by each respondent are shown in Table 2. The leading causes of injury was pulling, pushing or lifting (60.2%), followed by being struck or pushed against objects (13.3%) and a fall, a slip or a trip (9.4%). Although 100 falls, slips or trips were reported, only 12 were responsible for the severe injuries suffered by flight attendants. The commonest injuries were strains and sprains (52.3%). Burns and cuts accounted for 25% and 14% of the injuries respectively. Turbulence related injuries were reported by 38 (29.7%) of the flight attendants. Service trolley (24.2%) and baggage/luggage (14.8%) were the other common objects related to injuries. Upper limbs (44.5%) and the back (32%) were the commonest sites affected. Nearly one-third (31.7%) required time off from work as a result of the injury. The median time lost was three days (Inter Quartile range 2 to 5 days). Only one of the injured required more than two weeks for recovery. Two-thirds of the injured flight attendants admitted that the injury could have been prevented if they were more careful.

**Table 2:** Details of the severest injury reported by 128 Sri Lankan cabin crew members

	**N**	**(%)**
**Manner in which the injury occurred**		

Pulling, pushing or lifting	77	(60.2)

Being struck by or pushed against an object	17	(13.3)

Fall, slip or trip	12	(9.4)

Other	22	(17.2)

**Nature of injury**		

Strain/sprain	67	(52.3)

Burn	32	(25.0)

Cut	18	(14.1)

Contusion/bruise	7	(5.5)

Fracture/dislocation	2	(1.6)

Other	2	(1.6)

**Object/factors associated with injury**		

Turbulence	38	(29.7)

Service trolley	31	(24.2)

Handling baggage/luggage	19	(14.8)

Overhead bins	8	(6.3)

Other	30	(25.0)

**Part of the body affected**		

Upper limb	57	(44.5)

Back	41	(32.0)

Lower limb	12	(9.4)

Shoulder	9	(7.0)

Head/neck	7	(5.5)

Other	2	(1.6)

**Took time off to recover**		

Yes	40	(31.7)

No	86	(68.3)

### Risk factors for onboard injuries

In the uni-variate analysis, the population which has higher risk of injury were females not belonging to Moor/Malay ethnic group, with less than seven years of onboard experience, below the age of 30, with weight less than 56 kilograms and have not undergone ergonomics training. Height was not associated with onboard injuries in the uni-variate analysis and was not included in the multivariate model. All potential predictors of injury found to be significant in the uni-variate analysis at the level of *p *< 0.2 were entered into the multivariate model. After controlling for other factors, female flight attendants had 2.94 times higher risk (95% CI 1.2–7.2) of being injured than males. Irrespective of the sex, body weight less than 56 kilograms (OR 2.88, 95% CI 1.4–5.8) and with less than seven years of on board experience (OR 10.51, 95% CI 3.6–31.0) were factors associated with higher risk of injury. Unadjusted and adjusted odds ratios of potential predictors of onboard injury are shown in Table 3.

**Table 3:** Predictors of the outcome variable "any injuries" among 322 Sri Lankan cabin crew members with estimated unadjusted and adjusted odds ratios

	**Unadjusted**			**Adjusted**		
	**OR**	**(95% CI)**	p	**OR**	**(95% CI)**	p

Age below 30 years	6.95	4.13–11.7	< .001	1.24	0.38–4.04	.723

Female	2.67	1.57–4.54	< .001	2.94	1.19–7.23	.019*

Weight < 56 Kg	5.42	3.33–8.81	< .001	2.88	1.44–5.78	.003*

Duration of service < 7 years	10.38	6.13–17.59	< .001	10.51	3.56–30.99	< .001*

Not being a Moor	6.59	2.28–19.06	.001	15.11	4.76–48.01	< .001*

Trained in ergonomics	0.74	0.31–1.79	.003	0.8	0.39–1.62	.528

*significant at p < 0.05

### Prevalence of Backache

During the six months preceding the study, 180 (55.9%) flight attendants experienced backache at least once. Self reported severity of backache was mild – 26.6%, moderate – 65.6%, and severe – 7.8%. Out of 180 flight attendants who had backache, 138 (76.7%) experienced it regularly (at least once a month) and nine (5%) had constant backache (at least once a week).

## Discussion

This study revealed that the incidence of onboard injury among flight attendants was 795 per 1,000 person years (95% CI 748.3–836.5), which is very high. Further, higher rate of onboard falls, trips and slips were found in this study. More than half the sample had backache during the six months preceding the study, the majority had regular attacks.

The present study had several limitations. The sample was not a proper random sample, consisted of batches of flight attendants recruited in the same time period in different years. Differences in training procedures, could affect the outcome of the present study. The second limitation was *recall bias*. Reporting of injury is dependent on recall, which depend on individual characteristics, severity of injuries and impact of the injury on the person.

A moving aircraft has multiple complex dynamic forces, which could lead to falls, trips and slips. They were the third commonest reported event in compensation claims filed by flight attendants in the USA[10]. The present study found that 16% of flight attendants had faced at least one such incident during the period of the study.

Incidence of injury reported in different sources provides a wide range of figures. These rates were mostly based on compensation reports and claims. However, a study conducted in Canada using a questionnaire survey of 60 flight attendants reported 48% of flight attendants sustaining onboard injuries[11]. Lee et al, (2006) reported 97% of work related musculoskeletal symptoms during a period of one year among flight attendants[12]. In studies using mailed questionnaires, despite a low response rate, the tendency to over report the occurrence of injury was high. The present study observed a higher injury incidence of 795 per 1,000 person years than rates based on reports, but lower than observed in studies using mailed questionnaires.

The commonest type of injury reported in the present study was strains and sprains and is compatible with previous studies[5,10,11]. The cause of this injury is usually overextension of muscles and tendons while pulling, pushing or lifting objects. Turbulence was the underlying cause for the majority of the injuries. In a previous study, an evaluation of 92 turbulence reported injuries among 179 flight attendants showed an upward trend in turbulence related injuries during the years, 1992 to 2001 and was attributed to increased flying hours[3]. However, equipments such as Turbulence Prediction and Warning System developed by NASA is believed to be helpful in reducing these injuries[13].

Analysis of risk factors in the present study raises several the following important points. It is evident that female flight attendants are at higher risk to injury due to higher biological vulnerability. However, the observed risk factors could be interrelated. In usual conditions, the turnover of female flight attendants is higher compared to their male counter parts. Their experience is limited and they are usually the junior member among the flight attendants. All these factors could contribute to a higher risk of injury among them. However, even after adjusting for duration of service and other factors, they still remained more vulnerable than males. The observation the study made on the Moor/Malay ethnic group is difficult to describe. However, the wide confidence interval suggests that the estimates are not stable and needs further studies.

An important finding of the present study is that the injuries were not associated with ergonomic training. This is in contrast to all recommendations in occupational safety guidelines. Nevertheless, the quality and content of these reported ergonomic training has not been evaluated, and in Sri Lanka, trained individuals available for ergonomic training are scarce. The reported training might not done by an experienced person and may not be proper and professional ergonomic training.

## Conclusion

Work related injury and musculoskeletal problems pose a major health hazard to flight attendants in Sri Lanka. Females are at higher risk, and the majority of the injuries are reported as preventable injuries. Initial as well as ongoing training programs for flight attendants should include safety procedures and proper ergonomic training by qualified personnel and should be accompanied by promotion skills development concerning these specific health problems.

## Competing interests

The authors declare that they have no competing interests.

## Authors' contributions

ASB designed the study, collected, analyzed and interpreted data, and prepared the manuscript. DSD and TCA helped in analysis and interpretation of data and in the preparation of the manuscript. All authors read and approved the manuscript.

## Pre-publication history

The pre-publication history for this paper can be accessed here:


